# American Medicinal Plants

**Published:** 1882-09

**Authors:** 


### BOOK NOTICES, REVIEWS, ETC.

American Medicinal Plants. An illustrated and descriptive guide, etc. By C. F. Millspaugh, M. D., Binghamton, N. Y. Bœricke & Taefel: New York and Philadelphia. 1882.

The homœopathic profession is fast becoming rich in the literature of its therapeutic agents. First, Allen's Encyclopædia, with its great Index; next, the American Homœopathic Pharmacopæia, and now Millpaugh's beautiful plates, with descriptive text, of the American flora used in homœopathic practice.

"The author has in every case drawn and colored the plants represented in this work, *by his own hand, from the specimens as they stood in the soil*, making mathematically accurate drawings, and thus avoiding the misrepresentations of wilted individuals, or too highly-colored fancy pictures.

"The work will contain one hundred and eighty colored illustrations, and complete text of all the plants indigen[illegible] naturalized in the United States, arranged *generically* when bound according to the numerical order of the plates.

"The preface, a glossary of bot[illegible] terms, and complete index, will be given with the latest part."

Dr. Millspaugh's plates are beautiful, and have received the highest praise from those best competent to judge. It is to be a subscription work; price, one dollar per part. Let all subscribe for it.

